# Effect of ceramic thickness and cement shade on the final shade after bonding using the 3D master system: a laboratory study

**DOI:** 10.1002/cre2.22

**Published:** 2016-03-10

**Authors:** Javier Montero, Cristina Gómez‐Polo

**Affiliations:** ^1^ Department of Surgery, Faculty of Medicine University of Salamanca Campus Miguel de Unamuno 37007 Salamanca Spain

**Keywords:** Dental cement shades, dental ceramics, dental color, porcelain thickness, spectrophotometry

## Abstract

The final color of a ceramic restoration is influenced by both the ceramic thickness and the cement shade. This study aims to evaluate the color stability according to the 3D Master System of e.max ceramic discs after bonding with different shades of luting agents. A total of 120 e.max.Press 2M1 HT ceramic discs (60 discs of 1‐mm thick and 60 discs of 0.5 mm thick) and three different values of Variolink Veneer cement were used (−3, 0, +3) for the cementation process. An Easyshade compact device was used to measure color shade tabs, according to the 3D Master System, on the discs both before and after the cementation protocols. Bivariate and multivariate analyses were carried out with the spss v.21. After bonding with the different luting agents, only 30% remained as 2M1: specifically, 22% of the thinner discs and 37.3% of the thicker discs. In general, the effect of bonding increased the value and the chroma of the shade to a significant extent. Regression analyses revealed that the most significant predictor for all color parameters was cement shade, the thinner disc group bonded with −3 cement being the most unstable subgroup. According to the 3D Master System, the shade of the luting agent was the main predictor of the final color. However, the final color seems to be somewhat unpredictable, at least according to the modulating factors evaluated in the present study.

## Background

Color is undoubtedly one of the parameters with the greatest weight when patients judge the quality of their restorations, above all in the anterior region of the mouth (Gómez‐Polo et al. 2014). There has been increasing interest in the use of porcelain laminate veneers as aesthetic restorations because these combine high aesthetic appeal, patient satisfaction, and less‐invasive tooth preparation (Calamia and Calamia 2007). However, it should also be noted that less‐invasive tooth preparation means that the thicknesses of the porcelain veneers are reduced, which may cause an increase in translucency leading to an aesthetic problem unless the factors related to the cementing technique used are precisely controlled.

The final color of a ceramic restoration may be influenced by translucency, opalescence, fluorescence, surface texture and shape properties, the brand and batches of the porcelain used, the number of porcelain firings and the condensation technique, and also by the color, translucency, and the thickness of the underlying resin luting agent (Barghi and McAlister 2003; Blackman 1982; Dozic et al. 2003). In light of these variables, manufacturers have been prompted to direct their efforts towards accurately predicting the final color of restorations, and this is why there is an increasing variety of colors available for resin luting‐agent shades. It has recently been reported that both the type and shade of resin cement and the thickness and shade of the ceramic influence the resulting optical color of laminate restorations when a colorimeter is used before and after bonding (Turgut and Bagis 2013).

The 3D Master Toothguide follows the order that best matches the capabilities of the human eye in terms of value, chroma and hue and corresponds to the importance of these three color elements in obtaining an accurate shade match (Browning et al. 2009). Value is determined by the amount of white or black; chroma describes the amount of hue or saturation of a given color, and hue is associated with the dominant wavelength of a color.

Dentistry faces the challenge of quantifying, predicting, and controlling the color changes inherent to treatment with laminate veneers. Most investigators studying this topic (Öztürk et al. 2013; Turgut and Bagis 2011; Turgut et al. 2014) have computed color changes by means of electronic devices using the CIELAB system, owing to its international standardization and its objectivity. Nevertheless, the color coordinates (CIELAB system) form part of a language with which most dentists are not familiar. Hence, the method of tooth color choice most widely used in clinical dentistry is subjective, using tooth‐guides.

To date, it has not been possible to establish the extent to which the initial color of a laminate veneer can be modified by considering the color of the resin cement, as well as the thickness of the ceramic (Archegas et al. 2011; Alqahtani et al. 2012) before the restoration is rejected or repeated when it clearly differs from the color of the adjacent teeth. In addition, clinicians are well aware that if the original color of the laminate veneer is similar to that of the adjacent teeth, inadequate choice of the final cement color may lead to an unwanted color change. There is a lack of studies about the effects of different luting‐agent shades under different thicknesses of ceramic veneer materials on the final color based on the 3D Master system. The 3D Master Toothguide is divided into five groups according to value or lightness. The group with the highest value is represented by 1 and the darkest one by 5. Shade tabs are identified with two numbers and a letter. The first number corresponds to value, the letter corresponds to hue, and the last number to chroma. Letter L shows a higher quantity of yellow, letter R, a larger quantity of red, and letter M indicates an intermediate hue. As specified by the manufacturer, chroma covers from the lowest intensity (1) to the highest intensity (3). In this color system, each color is determined by a number from 1 to 5 (value), a letter (hue: L, M, R), and another number from 1 to 3 (chroma). For example, in the color code 2M1, the 2 represents value group, the M represents hue, and 1 represents chroma.

Based on our previous clinical observations, we hypothesized that ceramic thickness would be a more determinant factor than cement shade in the prediction of the final color of the restorations. We also expected that a neutral‐shade cement would not alter the initial ceramic color. Furthermore, cement thickness should also be directly correlated with the final color of such resin‐bonded restorations.

The null hypothesis was that the ceramic thickness, the cement shade, and the cement thickness are not able to significantly influence the color change after bonding. Thus, the aim of this in vitro study was to evaluate color change according to the 3D Master System of laminate veneers of 0.5 mm or 1 mm thickness after bonding with three shades of luting agents.

## Materials and Methods

One hundred and twenty ceramic discs simulating veneer thickness were prepared from A2 HT (high translucent) shades of IPS e.max Press (IvoclarVivadent, Schaan, Liechtenstein) of 0.5‐mm (60 discs) and 1.0‐mm thickness (60 discs) with a diameter of 8.0 mm. The ceramics were divided into three groups according to the shade (+3: high value, 0: translucent, −3: low value) of the Variolink Veneer luting agent from the same manufacturer. Thus, each of the six experimental subgroups contained 20 specimens (*n* = 20), that is, three distinct shades between both the thinner and thicker ceramic subgroups. The −3 and +3 shades are assumed to alter the final brightness value of the ceramic, that is, +3 high‐value pastes allow the restoration to appear lighter (more value or more whiteness), whereas low‐value −3 impart a darker or more yellowish character to the ceramic restoration. All specimens were placed on a middle‐grey background (middle grey is a tone that is perceptually about halfway between black and white on a value), with the ceramic surface always facing the measurement side.

All discs were of the all‐ceramic lithium disilicate type (IPS e.max.PressA2 HT). According to the manufacturer, the true color of the ceramic ingot was A2, this being equivalent to 2 M1 in the Master 3D system. The specimens were prepared according to the manufacturer's instructions. The thickness of the specimens was measured three times with a digital caliper (Electronic Digital Format CalliperIP54), and the specimen means were within the 0.50 ± 0.06 and 1.00 ± 0.01 mm range. The specimens were cleaned ultrasonically for 10 min before cementation and were divided into six thickness and Variolink Veneer shade groups (*n* = 20). We also used the digital caliper to estimate cement thickness.

A standardized protocol for color evaluation was adopted for all discs, and all color recordings were carried out in the same room under standardized lighting conditions (D65 daylight fluorescent tubes with a light intensity of 1500 lux). The spectrophotometer used was a Vita Easyshade Compact (Vita‐Zahnfabrik, Bad Sackingen, Germany). After the probe tip calibration, the Easyshade compact probe was placed in contact with and perpendicular to the middle of the top surface of the discs. All discs were recorded in the “Single Tooth” mode. The probe tip has 19 optic fibres measuring 1 mm in diameter. The outer ring is formed by 12 packets of fibres and is used to illuminate the 5 mm diameter of the probe tip (from 0° to 30°).

To analyze color change, the luting agents were bonded to the initially colored 2M1 ceramic discs. On each disc, the area designated for contact with the cement material was prepared with 5% hydrofluoric acid in gel applied for 1 min, rinsed with water for 30 sec, and dried with oil‐free air. Following this, a monocomponent silane was applied for 1 min and then dried for 3 sec with oil‐free air. Bonding was performed using Heliobond for the Variolink Veneers, following the manufacturer's instructions (Ivoclar Vivadent AG, Schaan, Liechtenstein).

Resin cements were applied directly from a syringe to the unglazed area of the ceramic surfaces. We used the same amount of cement for both discs thickness. Following this, a clean glass slide was placed onto the resin mixture and a manual pressure of approximately 500 grs was applied to it for 5 sec to simulate clinical conditions. Then, a light‐emitting diode was applied in direct perpendicular contact with the top surfaces (Bluephase, IvoclarVivadent, Liechtenstein) with a power of 1100 nW/cm^2^ for 40 sec. For all specimens, color was measured immediately after curing process using the Vita Easyshade compact, and the first result that coincided twice was recorded. This provided results coded according to the 3D‐Master System. To perform comparisons regarding the color change, we used the baseline color of all specimens as controls.

Bivariate and multivariate analyses were carried out to assess the influence of the thickness of the ceramic and the value and thickness of the cement on the resulting shade, using both paired non‐parametric (Wilcoxon Rank Tests) and parametric tests (such as Student's *t*‐tests for independent samples and step‐wise linear regression analyses). Step‐wise linear regression analysis was conducted to measure the relative weight of the independent variables in predicting the color parameters. All analyses were performed with the spss v.21 (Statistical Package for the Social Sciences, Chicago, IL).

## Results

Table 1 shows that after bonding, the real thickness of the ceramic disc increased significantly in both thickness subgroups, ranging between 150 and 300 µm on average. At baseline, the distributions of the Toothguide 3D Master parameters were almost identical (2M1). However, in both thickness groups, the effect of bonding reduced the value parameter significantly and increased the chroma of the shade. However, only among the thicker ceramic discs were significant differences in hue observed after bonding. Thus, after bonding, the discs were lighter. Table 1 shows that after bonding, half of the discs increased the value, from value = 2 to value = 1. The discs did not darken in any case. None of the discs had a final color belonging to value = 3. Regarding chroma, after bonding, most of the discs were assigned to chroma 1, both those in the thin disc group (71.7%) and those in the thick disc group (78.3%). However, in the thin discs, all the chroma groups were represented, whereas in the thick discs, only chromas of 1.5 (15%) and chroma 2 (6.7%) were obtained. With respect to hue, after bonding in all groups, a trend towards the M hue group was observed (ranging between 80% and 85%), and the deviation from this trend was above all towards the L hue group. Despite this, in the 0‐ to 5‐mm discs there was dics, although marginal, towards the R hue group, and this was not seen with the 1‐mm discs.

**Table 1:** Bivariate analyses of the effect of ceramic thickness on the VITA_3D MASTER parameters in the study sample (*n* = 120).

	Thin ceramic discs 0.5 mm thickness (*n* = 60)		Thick ceramic discs 1.0 mm thickness (*n* = 60)	
	Baseline	After bonding	Baseline	After bonding
	Mean (SD)	Mean (SD)	Mean (SD)	Mean (SD)
Real thickness (mm)	0.43(0.06)^a^	0.64(0.12)^a^	0.95(0.01)^a^	1.11(0.10)^a^
Cement thickness (mm)		0.29(0.12)*		0.16(0.12)*
3D‐Master parameters	*N*(%)	*N*(%)	*N*(%)	*N*(%)
value				
1	0(0%)^a^	30(50%)^a^	1(1.7%)^a^	28(46.7%)^a^
2	60(100%)^a^	30(50%)^a^	59(98.3%)^a^	32(53.3%)^a^
3	0(0%)	0(0%)	0(0%)	0(0%)
4	0(0%)	0(0%)	0(0%)	0(0%)
5	0(0%)	0(0%)	0(0%)	0(0%)
Hue				
L	1(1.7%)	9(15%)	0(0%)^a^	9(15%)^b^
M	59(98.3%)	48(80%)	60(100%)^a^	51(85%)^a^
R	0(0%)	3(5%)	0(0%)	0(0%)
Chroma				
1	59(98.3%)^a^	43(71.7%)^a^	60(100%)^a^	47(78.3%)^a^
1.5	0(0%)^a^	8(13.3%)^a^	0(0%)^a^	9(15.0%)^a^
2	0(0%)^a^	4(6.7%)^a^	0(0%)^a^	4(6.7%)^a^
2.5	1(1.7%)^a^	2(3.3%)^a^	0(0%)	0(0%)
3	0(0%)^a^	3(5.0%)^a^	0(0%)	0(0%)


* Significant results after independent *t*‐test analyses (*p*‐value <0.05)

a Significant differences within the standardized thickness subgroups after running the Wilcoxon Rank Tests to compare the baseline and the post‐bonding data distributions (*p* <0.01).

Table 2 shows that none of the discs was darkened after bonding. Among the thinner ceramic discs, hue did not differ significantly between the cement subgroups, but chroma was significantly greater (with more saturation) after bonding in the −3 luting‐agent shade group. Moreover, the final color was lighter after bonding only when 0 or +3 cement shades were used in both the thin and thick ceramic discs. By contrast, hue only changed significantly in the thicker discs bonded with the −3 luting agent towards a more yellowish shade. As a summary, the greatest change occurred in the value rather than in the hue or chroma parameters. In addition, the shade changes tend to be more marked in the thinner ceramic disks.

**Table 2:** Bivariate analyses of the effect of the value of the bonding cement on the VITA_3D MASTER parameters in each ceramic thickness group (*n* = 120).


	Thin ceramic discs 0.5 mm thickness (*n* = 60)						Thick ceramic discs 1.0 mm thickness (*n* = 60)					
Variolink veneer shade	−3 (*n* = 20)		0 (*n* = 20)		+3 (*n* = 20)		−3 (*n* = 20)		0 (*n* = 20)		+3 (*n* = 20)	
	Baseline	After bonding^1^	Baseline	After bonding	Baseline	After bonding	Baseline	After bonding	Baseline	After bonding	Baseline	After bonding
Value												
1	0(0%)	1(5%)	0(0%)^1^	17(85%)	0(0%)^1^	12(60%)^1^	0(0%)	2(10%)	1(5%)^1^	7(35%)^1^	0(0%)^1^	19(95%)^1^
2	20(100%)	19(95%)	20(100%)^1^	3(15%)^1^	20(100%)^1^	8(40%)^1^	20(100%)	18(90%)	19(95%)^1^	13(65%)^1^	20(100%)^1^	1(5%)^1^
3	0(0%)	0(0%)	0(0%)	0(0%)	0(0%)	0(0%)	0(0%)	0(0%)	0(0%)	0(0%)	0(0%)	0(0%)
4	0(0%)	0(0%)	0(0%)	0(0%)	0(0%)	0(0%)	0(0%)	0(0%)	0(0%)	0(0%)	0(0%)	0(0%)
5	0(0%)	0(0%)	0(0%)	0(0%)	0(0%)	0(0%)	0(0%)	0(0%)	0(0%)	0(0%)	0(0%)	0(0%)
Hue												
L	0(0%)	7(35%)	0(0%)	2(10%)	1(5%)	0(%)	0(0%)^1^	8(40%)^b^	0(0%)	1(5%)	0(0%)	0(0%)
M	20(100%)	10(50%)	20(100%)	18(90%)	19(95%)	20(100%)	20(100%)^1^	12(60%)^1^	20(100%)	19(95%)	20(100%)	20(100%)
R	0(0%)	3(15%)	0(0%)	0(0%)	0(0%)	0(0%)	0(0%)	0(0%)	0(0%)	0(0%)	0(0%)	0(0%)
Chroma												
1	20(100%)^1^	4(20%)^1^	20(100%)	20(100%)	19(95%)	19(95%)	20(100%)^1^	10(50%)^1^	20(100%)	19(95%)	20(100%)	18(90%)
1.5	0(0%)^1^	8(40%)^1^	0(0%)	0(0%)	1(5%)	0(0%)	0(0%)^1^	8(40%)^1^	0(0%)	1(5%)	0(0%)	2(10%)
2	0(0%)^1^	3(15%)^b^	0(0%)	0(0%)	0(0%)	1(5%)	0(0%)^1^	2(10%)^1^	0(0%)	0(0%)	0(0%)	0(0%)
2.5	0(0%)^1^	2(10%)^1^	0(0%)	0(0%)	0(0%)	0(0%)	0(0%)	0(0%)	0(0%)	0(0%)	0(0%)	0(0%)
3	0(0%)^1^	3(15%)^1^	0(0%)	0(0%)	0(0%)	0(0%)	0(0%)	0(0%)	0(0%)	0(0%)	0(0%)	0(0%)

1 Significant differences in the thickness and cement subgroups after running the Wilcoxon Rank Tests to compare the baseline and the post‐bonding data distributions (*p* <0.01).

The regression analyses showed that the most significant factor was the cement shade, which significantly affected all the final color parameters. The post‐bonding decrease in the value parameter would range between −0.44 and −0.26 units if a neutral luting agent shade were used instead of a −3 luting agent shade and similarly if a +3 were used instead of the neutral cement. The hue of the ceramic changed from L to M, in consonance with the cement shade. The post‐bonding chroma was significantly modulated by the cement shade but would also have been significantly increased if the thickness of the ceramic had been reduced. Nevertheless, even though all the potential modulatory variables had been introduced in the regression models, the final color parameters were only partially predicted, revealing that the final color is somewhat unpredictable (see corrected *R* ^2^ in footnotes of Table 3).

**Table 3:** Step‐wise linear regression models for predicting the final color parameters after including all the potentially related variables, that is, baseline parameter, thickness of the ceramic disc, cement thickness, and cement value (−3, 0, +3).

Models/parameters	Hypothesis contrast				CI−95%	
	B	Error	Standardized B	*p*‐value	Lower	Upper
Post‐bonding value^1^						
(Intersection)	1.5	0.04		0.000	1.4	1.6
Variolink Veneer shade (−3, 0, +3)	−0.4	0.05	−0.57	0.000	−0.44	−0.26
Post‐bonding hue^2^						
(Intersection)	1.9	0.04		0.000	1.8	1.9
Variolink Veneer shade (−3, 0, +3)	0.15	0.04	0.31	0.001	0.07	0.24
Post‐bonding chroma^3^						
(Intersection)	1.0	0.06		0.000	0.9	1.1
Variolink Veneer shade (−3, 0, +3)	−0.23	0.04	−0.43	0.000	−0.32	−0.15
Standardized ceramic thickness (1 mm as reference)	1.4	0.29	0.36	0.000	0.78	1.91


1 *F* = 57.34 gl = 1; *p* < 0.001; Corrected *R* ^2^ = 0.32.

2 *F* = 12.26. gl = 1; *p* = 0.001; Corrected *R* ^2^ = 0.09.

3 *F* = 27.89. gl = 2; *p* < 0.001; Corrected *R* ^2^ = 0.31.

Table 4 shows the changes that occurred in color according to the Master 3D system after cementing 2M1 discs of different thicknesses with different cement values. The Table does not include the two discs whose initial color (baseline) was not the 2M1 shade. At baseline, 98.3% of the discs were identified as 2M1, but after bonding with the different luting agents, only 30% persisted as 2M1. In the 0.5‐mm‐thick discs, after bonding, only 22% of the discs remained with the initial 2M1 color, while in the 1.0‐mm group, color stability was observed in 37.3% of the discs. Of the 20 thin discs cemented with 0 shade luting agent, 90% increased in value (value group 1), maintaining their hue (M group) and chroma (1 group), whereas this effect only occurred in 36.8% (*n* = 7) of the 1.0‐mm‐thick group. Moreover, 95% of the 1.0‐mm‐thick discs and 62.3% of the 0.5‐mm discs became lighter when cemented with the +3 shade luting agent. The greatest variability in colors after cementing was obtained for the 0.5‐mm ceramic discs cemented with −3 shade luting agent, observing values ranging between 1M2 up to 2M3, passing through tones of different hue, chroma, and value, although most of them ended at 1L1.5 (35%) or the original color of 2M1 (20%).

**Table 4:** Changes according to the 3D_Master guide of ceramic discs initially recorded as 2M1 (*n* = 118) after bonding depending on cement value and the ceramic thickness.

	Thin ceramic discs 0.5 mm thickness (*n* = 60)			Thick ceramic discs 1.0 mm thickness (*n* = 60)		
Variolink Veneer shade	−3 (*n* = 20)	0 (*n* = 20)	+3 (*n* = 19)	−3 (*n* = 20)	0 (*n* = 19)	+3 (*n* = 20)
1M1		18(90%)	11(57.9%)		7(36.8%)	17(85%)
1M2	1(5%)		1(5.3%)	2(10%)		2(10%)
2M1	4(20%)	2(10%)	7(36.8%)	10(50%)	11(57.9%)	1(5%)
2L1.5	7(35%)			8(40%)	1(5.3)	
2R1.5	1(5%)					
2R2.5	2(10%)					
2M2	2(10%)					
2M3	3(15%)					

## Discussion

Dentistry faces the challenge of quantifying, predicting, and controlling the color changes inherent to treatment with laminate veneers. This is because color is one of the most influential factors in patient acceptance of a tooth restoration and at the same time is very difficult to achieve. The clinical impact lies in the fact that if there is a mistake when choosing the shade tab of the 3D Master Toothguide, there will very probably be an unacceptable color difference from a clinical point of view. This fact causes an evident alteration in esthetic restorations, which may end in patient rejection (Gómez‐Polo et al. 2015). This is especially the case of single‐unit restorations in the anterior part of the mouth, owing to the difficulty involved in achieving the same color as the adjacent natural teeth. This study aims at assessing the chromatic changes occurring after the cementation process on the basis of the thickness of the ceramic and the shade gradient of the luting agents used according to the 3D Master system. We report our results using 3D Master codes because this color system is widely known among most clinicians and is easier to understand, although it is not as accurate as the standardized CIELAB assessments, used in most studies (Chen et al. 2015; Öztürk et al. 2013; Turgut and Bagis 2011; Turgut and Bagis 2013; Turgut et al. 2014). In fact, our results are in full agreement with those studies, whose authors concluded that the shade of resin cements and the thickness and shade of the ceramic used influenced the resulting optical color of laminate restorations. We believe that most of the color changes observed here would be detectable to the human eye, mainly when a dark cement was used to bond a thin restoration (Table 4). However, it should be addressed in future studies.

*Try‐in* pastes may be used as an approximation of the final color of the restoration, but they are unable to reliably represent the exact final color, because they are not affected by the unavoidable polymerization‐related color changes. Future studies should focus on analyzing the color differences between the try‐in pastes and the corresponding luting agents.

Another limitation of this study concerns the middle‐grey background against which all the color measurements were made. The influence of background on color is a somewhat controversial topic because there is little consensus in the literature. However, in this regard, grey and black backgrounds have been found to simulate intra‐oral environments better than white ones (Ardu et al. 2014). Future works will be designed to replicate the research but using different backgrounds in order to better address this confounding factor. With respect to sample size, we observed that a cell size of 20 discs was adequate for the study purpose given the small dispersion of the data. Many of the works addressing this issue have used an “in vitro” sample similar in size or even smaller (Kilinc et al. 2011; Kürklü et al. 2013; Niu et al. 2014) than that used in this study. Turgut et al. 2013 prepared 392 discs, but the effective cell size was clearly smaller than ours, because those authors combined 4 ceramic shades, 2 thicknesses, and 13 shades of resin cements.

The cement thickness observed in this study exceeds the one recommended by the manufacturer (15 µm). In a try to simulate clinical conditions, the cement thickness was not standardized using spacers. In fact, in spite of using the same amount of luting cement for both ceramic thicknesses, the cement thickness for the 0.5‐mm discs was almost twice the thickness of the 1 mm discs. This may be a technical‐related event, that is, the operator should exert a higher manual pressure onto the thicker discs than onto the thinner discs, because of the higher weakness of the latter. Anyway, because we were well aware that this continuous variable should significantly modulate the color changes, it was introduced as a potential predictor within the linear regression models (Table 3), resulting in a non‐significant effect (Table 3), after computing the modifying effect of the major predictors (cement shade and ceramic thickness.

The cement thickness obtained in this study was the result of a luting process carried out by a single operator, and hence, the variability in concentration and meticulousness during the procedural steps may have partially altered our results. However, the magnitude of the marginal fit reported here is clearly lower than that reported in several clinical trials (Niu et al. 2014; Celik et al 2002; Yeo et al. 2003) in which a gap below 120 µm was considered the maximum clinically acceptable marginal misfit.

Several authors (Chang et al. 2009; Kilinc et al. 2011; Kürklü et al. 2013; Niu et al. 2014; Turgut and Bagis 2011) agree that the variables analyzed (ceramic thickness, luting agent thickness, ceramic systems, luting agent shade) influence the final shade of restorations to a significant extent. The differences emerge when trying to set up the degree of influence of each variable. Contrary to our initial hypothesis, we found that although ceramic thickness modulated the final shade, the major predictor was cement shade (Table 3). Thus, null hypothesis was rejected.

In this study, the +3 y −3 shade luting agents were chosen because they are extreme shades. In this way, it is possible to predict the upper and lower thresholds with respect to the changes in shades expected after cementing. Our results demonstrate that the shade of the luting agent affects not only the shade value but also the chroma and the hue. According to the manufacturer, the zero luting agent affects value only slightly or not at all. The results described here show that shade became lighter (increase value) in 85% of the cases with thicknesses of 0.5 mm and in 35% of the cases with thicknesses of 1 mm. Hence, special caution should be exercised when cementing thin veneers (0.5 m) with 0 neutral shade luting agents because the value of the final shade would be increased. By contrast, the difficulty involved in darkening or decreasing the final lightness of restorations, even with minimum thicknesses, using the −3 Variolink luting agent shade should be noted, as reported by Chang et al. 2009.

These observations should be taken with caution, because cement thickness, which is directly correlated with the shade of a resin‐based cement, was almost double in the 0.5‐mm discs than in the 1.0‐mm discs (Table 1). However, the regression models did not identify cement thickness as a significant shade predictor when other stronger predictors were included (such as cement shade or ceramic thickness).

Furthermore, the shade differences among the different shade tab pairs (for example, between 2M1 and 1M1 or between 2M1 and 2L1.5) were clinically perceptible, exceeding the dentistry threshold of ΔE_ab_^*^ 2.6 units (Douglas et al. 2007). Emphasis should also be placed on the low predictability of the final post‐bonding shade after monitoring several of the factors considered essential to date, such as the thickness of the ceramic veneer and the shade and thickness of the luting agent (Table 3).

It should be taken into account that the shade changes reported here do not necessarily have to be homogeneous if the study is reproduced with another baseline ceramic shade or a ceramic from another manufacturer. For example, Leucite‐reinforced feldspathic ceramics (IPS Empress, Ivoclar Vivadent, Liechtenstein) have generally been found to be less yellow and darker than glass‐infiltrated aluminum oxide (In ceram, Vita Zahnfabrik, Germany) (Lee et al. 2007; Uludag et al. 2007).

Regarding ceramic thickness, in consonance with most authors, we conclude that the thinner the ceramic, the greater the influence of the underlying shade (Chang et al. 2009; Turgut and Bagis 2011; Turgut and Bagis 2013; Vichi et al. 2009).

It would be interesting to perform further studies with different ceramic shades, with different luting agent shades from different manufacturers and with different ceramic thicknesses and then measure the results according to the Vitapan classical and 3D Master systems.

## Conclusions

Although ceramic thickness modulated the chroma of the final shade of the laminates, the major predictor was cement shade, which is able to alter all the shade parameters to a significant extent.

## Conflict of Interest

The authors declare that they have no conflict of interest.
